# Retraction Note: The BET inhibitor I-BET762 inhibits pancreatic ductal adenocarcinoma cell proliferation and enhances the therapeutic effect of gemcitabine

**DOI:** 10.1038/s41598-023-48298-9

**Published:** 2023-11-28

**Authors:** Fang Xie, Mei Huang, Xiansheng Lin, Chenhai Liu, Zhen Liu, Futao Meng, Chao Wang, Qiang Huang

**Affiliations:** 1 Department of General Surgery, Anhui Provincial Hospital, No. 17, Lujiang Road, Hefei, Anhui Province China; 2 Anhui Province Key Laboratory of Hepatopancreatobiliary Surgery, Hefei, China; 3 Anhui Medical University Affiliated Provincial Hospital, No. 9, Lujiang Road, Hefei, Anhui Province China

Retraction of: *Scientific Reports* 10.1038/s41598-018-26496-0, published online 25 May 2018

The Editors have retracted this Article. After the publication of this Article it was brought to the Editors' attention that some of the data appear to overlap with data in other articles with different authors, where they are attributed to different experiments. Specifically:

- In Figure 2A images of scratch wound assays in BxPC-3 and Panc-1 cells in control and I-BET762 groups appear to overlap with data described in^1,2^.
- In Figure 2D images of colony formation assays in BxPC-3 and Panc-1 cells in control and I-BET762 groups appear to overlap with data described in^3–6^.
- In Figure 3A the images of sphere formation assays appear to overlap with those described in^7^.

The Editors reached out to the Authors to request raw data. The Authors were unable to provide the data and the concerns remain unresolved. The Editors therefore no longer have confidence in the results presented in this Article. All Authors agree to this retraction.
